# Compatibility and synergistic interactions of fungi, *Metarhizium anisopliae*, and insecticide combinations against the cotton aphid, *Aphis gossypii* Glover (Hemiptera: Aphididae)

**DOI:** 10.1038/s41598-022-08841-6

**Published:** 2022-03-22

**Authors:** Ahmad Nawaz, Fatima Razzaq, Amna Razzaq, Muhammad Dildar Gogi, G. Mandela Fernández-Grandon, Muhammad Tayib, Muhammad Ahsin Ayub, Muhammad Sufyan, Muhammad Rafiq Shahid, Mirza Abdul Qayyum, Muhammad Naveed, Anam Ijaz, Muhammad Jalal Arif

**Affiliations:** 1 Department of Entomology, University of Agriculture, Faisalabad, Pakistan; 2 Department of Zoology, Wildlife and Fisheries, University of Agriculture, Faisalabad, Pakistan; 3 Natural Resources Institute, University of Greenwich, Central Avenue, Chatham Maritime, Kent ME4 4TB UK; 4 Rice Research Station, Bahawalnagar, Punjab Pakistan; 5 Cotton Research Institute (CRI), Multan, Punjab Pakistan; 6 Institute of Plant Protection, Muhammad Nawaz Sharif University, Multan, Pakistan; 7 Institute of Soil and Environmental Sciences, University of Agriculture, Faisalabad, Pakistan

**Keywords:** Biological techniques, Plant sciences

## Abstract

Aphids are major pests affecting cereals, vegetables, fruit, forestry and horticultural produce. A multimodal approach may be an effective route to controlling this prolific pest. We assessed the individual and combined effect of eight insecticides and the entomopathogenic fungi, *Metarhizium anisopliae* (Metschin.) against the cotton aphid, *Aphis gossypii* Glover (Hemiptera: Aphididae), under laboratory conditions. Six of the insecticides tested were found to be highly compatible (flonicamid, imidacloprid, nitenpyram, dinotefuran, pyriproxyfen and spirotetramat), showing positive integration with the fungus and were selected for bioassays. The combination mixtures (1:1 ratio of *M. anisopliae*: insecticide) were significantly more toxic to *A. gossypii* than individual treatments. Maximum mortality (91.68%) of *A. gossypii* was recorded with combination of flonicamid and *M. anisopliae* (2.4 × 10^6^ cfu/ml) 72 h after application. While minimum mortality (17.08%) was observed with the individual treatment of *M. anisopliae* (2.4 × 10^6^ cfu/ml). The insecticides revealed toxicity consistent with their compatibility with *M. anisopliae,* ranking for efficacy exactly as they did for compatibility. In addition, the synergy factor (SF) and co-toxicity coefficient (CTC) values indicated synergistic interactions at different time intervals. The synergistic efficacy revealed the potential of fungus-insecticide integration against sucking insect pests.

## Introduction

Aphids are small sap-sucking insects. Among the 5000 described species, 450 aphid species cause intense damage to crop and ornamental plants around the world^1^. They are distributed globally but most commonly found in temperate zones where species diversity is also much higher compared to the tropics^2^. Aphids are considered serious pests because they reach a high population density and can develop resistance to insecticides in a short period of time^3,4^. The cotton aphid, *Aphis gossypii* Glover (Hemiptera: Aphididae), is a highly polyphagous pest. It causes serious damage like leaf curling, leaf deformation and transmits at least 76 viral diseases including potyvirus, cucumber mosaic virus and zucchini yellow virus to a wide range of crops^5^. Aphid nymphs and adults deplete photo assimilates through their feeding and devitalize the plant in the process^6^). Aphids also secrete honeydew which attracts black sooty mould that stains cotton fiber and blocks photosynthesis. The honeydew also causes sticky cotton during mechanical harvesting, ginning, and processing^7^. Several control measures including host plant resistance, cultural, biological and chemical control are utilized to keep the pest population below economic injury level^8^. Sucking insect pests like aphids and whiteflies can be controlled by using neonicotinoids^9^. Neonicotinoids act as inhibitor on nicotinic acetylcholine receptors in the central nervous system^10^. The intensive use of insecticides to control cotton aphids has led to populations that are now resistant to several classes of insecticides^11^. In addition, pesticides can cause serious problems of environmental contamination and adverse effects on beneficial insects such as bee populations^12–14^. Biopesticides offer a route to protecting the crop while reducing the reliance on synthetic insecticides^15^. Entomopathogenic fungi (EPF) have been found to be effective as a biopesticide^16^ and have potential to minimize the target pest populations on multiple crops^17–20^. Moreover, 750 species of EPF are known to inoculate insect pests^21^. One commonly used entomopathogenic fungi is *Metarhizium anisopliae* (Metschin.), which has been shown to be effective for control against 200 insect species^22^ including *Aphis gossypii*^23,24^). More than 150 insect biocontrol products based on fungal entomopathogens have been commercialized with over 75% of these products based on the hypocrealean fungi *M. anisopliae*, *Beauveria bassiana*, *Isaria fumosorosea*, and *B. brongniartii*^16^, however this number is expected to have increased since the last major market evaluations were conducted. Entomopathogenic fungi are generally considered slow-acting, taking longer than conventional methods to achieve sufficient insect mortality. The technique of combining EPF into a management strategy with faster-acting materials may be the solution to this problem. The synergistic action of mycoinsecticides with chemical insecticides can increase mortality and reduce the time until death in insects^25–28^. The combined use of fungal pathogens and the full, or reduced, dose of chemical insecticides is a promising pest-control option. The application of synergists can effectively enhance the cost-effectiveness and eco-friendliness of insecticides by reducing the required quantity and extending the residual activity. By attacking the pest through a different mode of action, they are equally important as an alternative for resistance management. The data is lacking regarding the compatibility of EPF with insecticides and synthetic insecticide combinations with mycoinsecticides are rarely evaluated against aphids. In this study we gauge the compatibility of different insecticides with *M. ansopliae* and assess their toxicity to a prominent aphid pest.

## Materials and methods

### *Metarhizium anisopliae* culture

Potato Dextrose Broth (PDB) media was used ^56^ in a 1000 ml Erlenmeyer flask and autoclaved at 121 °C for 20 min as previously described^29^. A disc of the cultured fungi approximately 5 mm in diameter was taken from its Petri dish and added into the prepared media under a laminar air flow chamber and kept at 25 ± 1 °C for 5 days before being transferred to a shaking incubator (Firstek Scientific, Tokyo, Japan) at 180 rpm for 48 h at 28 ± 1 °C. An optical density of 0.5 was measured with an OD meter (BIOLOG MODEL-21907; BIOLOG INC.) at λ 600 nm. This was achieved by dilution to maintain uniform conidia density (10^6^ CFU mL^–1^) prior to application. Inoculum and saline buffer (0.85% NaCl w/v) at ratios 1:9 and 2:18 were mixed to prepare *M. anisopliae* suspensions containing 10^6^ CFU mL^−1^. To achieve these populations, OD 0.4 and 0.3 samples were adjusted prior to application.

### Insecticides compatibility with *M. anisopliae*

To assess compatibility, the effect of different insecticides (flonicamid, imidacloprid, nitenpyram, dinotefuran, pymetrozine, pyriproxyfen, spirotetramat and matrine) on the radial growth of *M. anisopliae* was evaluated. The recommended field doses of insecticides were added to potato dextrose agar (PDA) in an Erlenmeyer flask before solidification. After mixing thoroughly, the media was transferred to Petri dishes and with gentle shaking allowed to solidify. Using a micropipette, *M. anisopliae* formulation (2.4 × 10^6^ CFU mL^–1^) was inoculated in each petri dish on media. The Petri dishes were sealed and placed in an incubator maintained at 25 ± 1 °C, 80 ± 5% relative humidity. The media without insecticide (Tween 80, 0.05%) was used as a control treatment. Fungal colony diameter was calculated after 3 days of inoculation using Vernier calipers. Treatment groups were compared to growth observed in the control to evaluate the potential impact of the insecticide on colony development.

### Toxicity assessment of insecticide—*M. anisopliae* combinations

To examine the interaction effect of *M. anisopliae* with insecticides against *A. gossypii*, six insecticides (Flonicamid, imidacloprid, nitenpyram, dinotefuran, pyriproxyfen and spirotetramat) that exhibited good compatibility with *M. anisopliae* were selected. A population of cotton aphids was collected from the Entomological Research Farm, Department of Entomology, University of Agriculture, Faisalabad, Pakistan. Serial dilutions of the *M. anisopliae* isolate, insecticide and mixture (*M. anisopliae* + insecticide) were prepared for each treatment (Table 1). After sterilization with sodium hypochlorite (0.5% v/v), detached cotton leaves were washed three times with distilled water, air dried and placed on 1.5% agar (non-nutritive) in 90 × 20 mm^2^ plastic Petri dishes. The 1.5% agar supplied moisture to maintain relative humidity during the test. Around 25 aphids (mixed adult and nymph population) were collected and allowed to settle for 1 day before treatment. A topical spray method was used to treat the aphids with individual and combined applications of insecticides and *M. anisopliae* applied using a hand atomizer (WIRELESS ATOMIZER SPRAYER, A7-01). Three replicates were completed for each treatment. Mortality data were recorded 24, 48 and 72 h post treatment.

**Table 1:** Insecticides and entomopathogenic fungi individual and combined application with different doses used for laboratory bioassays.

S. no	Treatment	Concentrations		
	Individual and combined molecules	Sub lethal concentration (C1)	Lethal concentration (C2)	Super lethal concentration (C3)
T_1_	Flonicamid	0.03%	0.06%	0.12%
T_2_	Imidacloprid	0.125%	0.25%	0.5%
T_3_	Nitenpyram	0.02%	0.04%	0.08%
T_4_	Dinotefuran	0.0375%	0.075%	0.15%
T_5_	Pyriproxyfen	0.23%	0.45%	0.9%
T_6_	Spirotetramat	0.062%	0.125%	0.25%
T_7_	*M. anisopliae*	2.4 × 10^6^ cfu/ml		
T_8_	Flonicamid + *M. anisopliae*	0.03% + 2.4 × 10^6^ cfu/ml	0.06% + 2.4 × 10^6^ cfu/ml	0.12% + 2.4 × 10^6^ cfu/ml
T_9_	Imidacloprid + *M. anisopliae*	0.125% + 2.4 × 10^6^ cfu/ml	0.25% + 2.4 × 10^6^ cfu/ml	0.5% + 2.4 × 10^6^ cfu/ml
T_10_	Nitenpyram + *M. anisopliae*	0.02% + 2.4 × 10^6^ cfu/ml	0.04% + 2.4 × 10^6^ cfu/ml	0.08% + 2.4 × 10^6^ cfu/ml
T_11_	Dinotefuran + *M. anisopliae*	0.0375% + 2.4 × 10^6^ cfu/ml	0.075% + 2.4 × 10^6^ cfu/ml	0.15% + 2.4 × 10^6^ cfu/ml
T_12_	Pyriproxyfen + *M. anisopliae*	0.23% + 2.4 × 10^6^ cfu/ml	0.45% + 2.4 × 10^6^ cfu/ml	0.9% + 2.4 × 10^6^ cfu/ml
T_13_	Spirotetramat + *M. anisopliae*	0.062% + 2.4 × 10^6^ cfu/mll	0.125% + 2.4 × 10^6^ cfu/ml	0.25% + 2.4 × 10^6^ cfu/m
T_14_	Control	Water		

### Determination of synergistic effect

The toxicity of combined and isolated treatments was calculated based on LC_50_ and LC_90_ of insecticides and combination treatments with EPF using probit analysis. The co-toxicity coefficient^30^ and synergy factor^31^ for mixed formulation were calculated utilizing the LC_50_ and LC_90_ identified for each treatment.

$$\text{Co-toxicity} \; \text{coefficient }=\frac{\mathrm{Toxicity \; of \; insecticide } \, \left(\mathrm{alone}\right)}{\mathrm{Toxicity \; of \; insecticide \; with \; fungal \; extract}}\times 100$$

$$\text{Synergy} \; \text{ factor} \; (\text{SF}) = \frac{\mathrm{Toxicity \; of \; insecticide } \, \left(\mathrm{alone}\right)}{\mathrm{Toxicity \; of\; insecticide \;with \; fungal \; extract}}$$

Within this system, a SF value > 1 indicates synergism and an SF value < 1 indicates antagonism^32,33^.

### Statistical analysis

Percentage mortality of aphids was calculated by Abbot’s Formula^34^. The experiment was carried out under controlled condition inside the incubator (POL-EKO_APARATURA SP.J. S02ADF 180665) and collected data were checked for normality and homogeneity of variance using Shapiro–Wilk test. The P value obtained was larger than probability value of 5% which indicated that distribution of data was normal. Mortality data were recorded daily after treatment and analyzed using the Statistix software version 8.1. Percentage corrected mortality data were analyzed by main effects one way ANOVA through Multivariate General Linear Model (MGLM) Technique^35^, using a STATISTICA software version 10.0 to determine the parameters of significance and mean values for different treatments and followed by a Tukey’s honestly significant difference (HSD) test with significant differences recognized when p < 0.05^36^. The LC_50_, LC_90_, chi-square and confidence interval values for each extract were also calculated by Probit analysis using the Minitab Statistical Program^37^. Regression between aphid’s mortality and concentrations of insecticides was also established, using linear regression and Pearson correlation analysis at 5% level of probability. Scattered diagrams for concentration of each insecticides (alone or in combination) and mortality of aphid were also drawn to construct fitted simple regression line of mortality on concentrations.

## Results

### In vitro study on compatibility of insecticides with *M. anisopliae*

**Figure 1:**
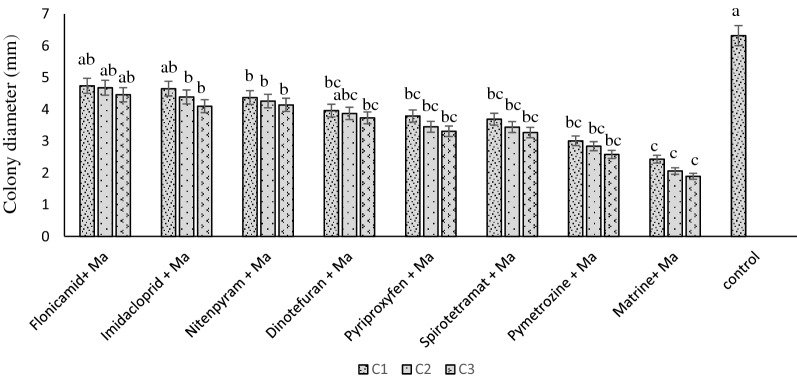
*M. Metarhizium anisopliae* compatibility test of eight different insecticides at different concentrations. The six insecticides with better compatibility (great colony growth) were chosen for toxicity bioassays. Letters above the bars indicate differences between treatments as determined by ANOVA followed by Tukey HSD. Those not sharing a letter are significantly different (p < 0.05).

Effects of the insecticides on *M. anisopliae* vegetative growth showed that all tested formulations significantly inhibited the fungal growth. However, insecticides did not all inhibit *M. anisopliae* growth to the same extent. The greatest radial growth of the fungi with any insecticide treatment was observed with flonicamid with a colony diameter of 4.74 mm at the lowest concentration. The mean diameters of colonies based on 3 replicates were 4.65, 4.37, 3.96, 3.79, and 3.69 mm for imidacloprid, nitenpyram, dinotefuran, pyriproxyfen, and spirotetramat respectively. The pymetrozine and matrine treatments led to the lowest radial growth (Fig. 1).

### Efficacy of treatments alone and in combination against cotton aphid

**Figure 2:**
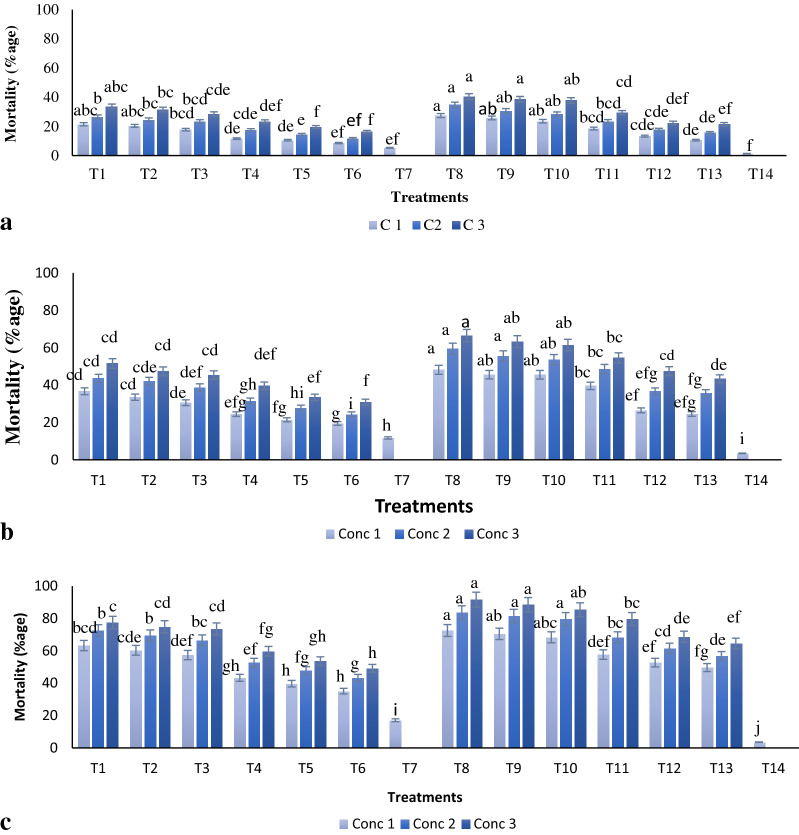
(**a**) Percentage mortality of cotton aphid 24 h post individual and combined treatment applications. Letters above the bars indicate differences between treatments as determined by ANOVA followed by Tukey HSD. Those not sharing a letter are significantly different (p < 0.05). The combined applications show significantly greater mortality than individual treatments and control. (C1 (F = 23.0; df = 13, 28; p < 0.000), C2 (F = 37.1; df = 13, 28; p < 0.0000), C3 (F = 75.0; df = 13, 28; p < 0.0001). (**b**) Percentage mortality of cotton aphid 48 h post individual and combined treatment applications. Letters above the bars indicate differences between treatments as determined by ANOVA followed by Tukey HSD. Those not sharing a letter are significantly different (p < 0.05). The combined applications show significantly greater mortality than individual treatments and control. (C1 (F = 93.1; df = 13, 28; p < 0.000), C2 (F = 163; df = 13, 28; p < 0.0000), C3 (F = 80.8; df = 13, 28; p < 0.0001). (**c**) Percentage mortality of cotton aphid 72 h post individual and combined treatment applications. Letters above the bars indicate differences between treatments as determined by ANOVA followed by Tukey HSD. Those not sharing a letter are significantly different (p < 0.05). The combined applications show significantly greater mortality than individual treatments and control. (C1 (F = 173; df = 13, 28; p < 0.000), C2 (F = 288; df = 13, 28; p < 0.0000), C3 (F = 321; df = 13, 28; p < 0.0001).

**Figure 3:**
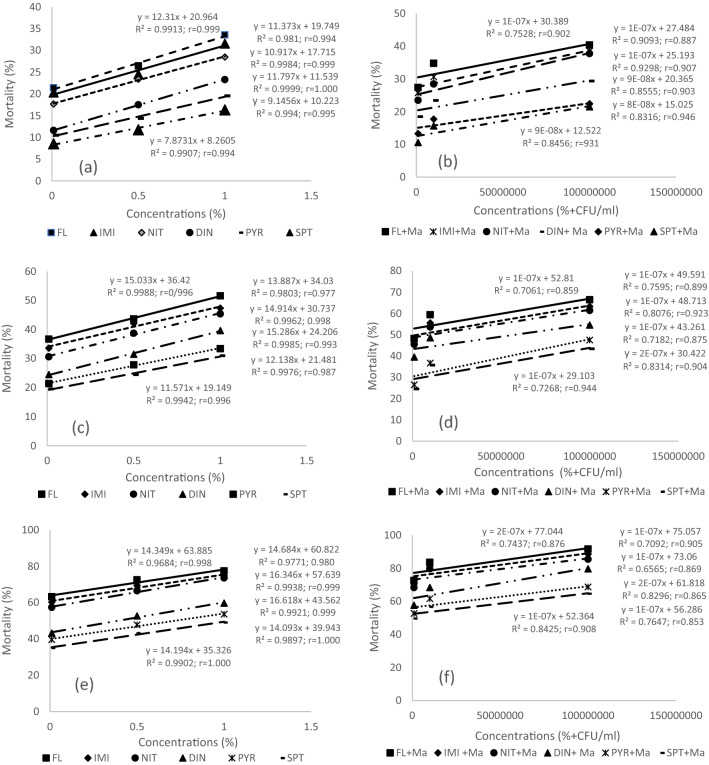
Correlation coefficient (r), linear regression equation (Ŷ = bx ± a), coefficient of determination (100 R^2^) andscatter plot showing a fitted simple regression lines of Ŷ (% mortality of *Aphis gossypii* in laboratory conditions) on X (concentration of insecticides alone and in combinations with *Metarhizium anisopliae*).

Percentage mortality of the cotton aphid after 24, 48 and 72 h post treatment were found significantly different (Fig. 2). *M. anisopliae* alone was least effective among all treatments, leading to 5.26, 11.76 and 17.08% mortality after 24, 48 and 72 h post exposure respectively. All insecticide-only treatments showed dose and time dependent toxicity. Flonicamid was most toxic followed by imidacloprid, nitenpyram, dinotefuran, pyriproxyfen, and spinotetramat presented at the lower doses. The combination mixtures of *M. anisopliae* and insecticides were significantly more toxic than individual treatments. The combined application of *M. anisopliae* with flonicamid exhibited the greatest mortality in *A. gossypii* after 72 h (91.68%), followed by mixtures of the EPF with imidacloprid (88.59%), Nitenpyram (85.45%), Dinotefuran (79.69%), Pyriproxyfen (68.73%), and Spirotetramat (64.63%) (Fig. 2c). The correlation coefficient values (r) demonstrate a positive correlation with mean percent mortality of the pest (Fig. 3).

### Synergetic effects of *M. anisopliae* and insecticides on *A. gossypii*

The LC_50_ and LC_90_ values of each insecticide and their mixture with *M. anisopliae* were inversely proportional with time. These values were used to determine the SF (Tables 2, 3, 4). Overall, it was observed that LC_50_ and LC_90_ values were lower in combination treatments than individual applications for the insecticides despite half the insecticides studied indicating an antagonistic effect with the EPF at the initial 24 h point.

**Table 2:** Toxicity of insecticides (Flonicamid and Imidacloprid) with and without *M. anisopliae* for *A. gossypii.*

Teatments	Ratio	Exposure period (hours)	Regression equation	Chi-square	LC_50_ ± SE (fiducial limits) (ppm)	SF	CTC	Type of action	LC_90_ ± SE (fiducial limits) (ppm)	SF	CTC	Type of action
Flonicamid		24	y = 133x + 17.85	0.008	0.439 ± 0.415 (0.161–13.852)	–	–	–	7.610 ± 0.747 (0.741–35.010)	–	–	–
		48	y = 160.67x + 32.74	0.150	0.122 ± 0.052 (0.071–17.320)	–	–	–	5.450 ± 0.253 (0.582–10.30)	–	–	–
		72	y = 147.24x + 60.8	0.250	0.010 ± 0.007 (0.00–0.04)	–	–	–	0.451 ± 0.383 (0.170–1335)	–	–	–
Flonicamid + *M. anisopliae*	1:1	24	y = 136.95x + 24.58	0.091	0.2173 ± 0.118 (0.112–56.531)	2.020	202.020	Synergistic	3.500 ± 0.875 (0.563–3378)	2.174	217.400	Synergistic
		48	y = 190.76x + 44.64	0.124	0.030 ± 0.011 (0.003–0.052)	4.066	406.660	Synergistic	0.943 ± 0.166 (0.280–6349)	5.797	579.710	Synergistic
		72	y = 201x + 68.512	0.006	0.008 ± 0.004 (0.0006–0.010)	1.250	125.000	Synergistic	0.100 ± 0.025 (0.070–0.261)	4.500	450.000	Synergistic
Imidacloprid		24	y = 29.737x + 16.8	0.011	1.933 ± 0.833 (0.700–20.021)	–	–	–	29.460 ± 5.374 (3.040–36.550)	–	–	–
		48	y = 34.457x + 30.97	0.151	0.611 ± 0.303 (0.331–20.652)	–	–	–	24.261 ± 4.920 (2.530–89.100)	–	–	–
		72	y = 36.354x + 57.61	0.110	0.040 ± 0.0381 (0.000–0.100)	–	–	–	3.271 ± 0.405 (0.900–16.590)	–	–	–
Imidacloprid + *M. anisopliae*	1:1	24	y = 33.777x + 21.781	0.029	1.400 ± 0.218 (0.561–63.011)	1.378	137.800	Synergistic	35.81 ± 8.585 (3.080–91.351)	0.822	82.261	Antagonistic
		48	y = 44.914x + 41.700	0.040	0.180 ± 0.0480 (0.031–0.270)	3.381	338.880	Synergistic	4.850 ± 5.641 (1.310–84,150)	5.002	500.200	Synergistic
		72	y = 45.263x + 67.020	0.063	0.031 ± 0.021 (0.001–0.070)	1.333	133.300	Synergistic	0.590 ± 0.193 (0.380–2.720)	5.542	554.231	Synergistic

*CTC* co-toxicity coefficient, *SF* synergy factor.

The LC_50_ of flonicamid against cotton aphid was 0.439 ppm at 24 h and 0.010 ppm at 72 h. The LC_90_ was 7.61 ppm at 24 h and 0.383 ppm at 72 h. The mixture of flonicamid with *M. anisopliae* showed synergistic interaction against *A. gossypii* (Table 2), dropping those values significantly for both 24 h (LC_50_ = 0.2173, LC_90_ = 3.50) and 72 h (LC_50_ = 0.008, LC_90_ = 0.10) mortality counts. The time dependent co-toxicity coefficient (CTC) oscillated from 202.02 to 125 and 217.4 to 450 for LC_50_ and LC_90_ respectively. The SF of the combination treatment varied at different time points but remained above 1 for both LC_50_ and LC_90_. Imidacloprid showed antagonistic interaction with *M. anisopliae* for LC_90_ after 24 h, however, thereafter showed a synergistic interaction (Table 2). At 72 h of exposure, CTC (133.3) and SF (1.33) values were reduced for LC_50_ while they increased to 554.23 and 5.542 for LC_90,_ respectively.

LC_50_ values of 0.21, 0.03 and 0.004 ppm and LC_90_ value of 3.80, 1.20 and 0.12 ppm after 24, 48 and 72 h respectively was seen for the nitenpyram with *M. anisopliae* combination (Table 3). These corresponded to CTC values for the LC_50_ of 187.6, 353.33 and 225 and for LC_90_ values of 170.7, 188.33 and 375 for 24, 48 and 72 h post treatment respectively. The SF suggested a synergistic interaction at for all time points assessed (Table 3).

**Table 3:** Toxicity of insecticides (Nitenpyram and Dinotefuran) with and without *M. anisopliae* for *A. gossypii.*

Teatments	Ratio	Exposure period (hours)	Regression equation		Chi-square	LC_50_ ± SE (fiducial limits) (ppm)	SF	CTC	Type of action	LC_90_ ± SE (fiducial limits) (ppm)	SF	CTC	Type of action
Nitenpyram		24	y = 172.61x + 15.151		0.100	0.394 ± 0.433 (0.121–3.030)	–	–	–	6.490 ± 6.325 (0.531–8.120)	–	–	–
		48	y = 234.82x + 27.282		0.090	0.106 ± 0.048 (0.061–15.420)	–	–	–	2.260 ± 3.899 (0.360–5040)	–	–	–
		72	y = 256.54x + 53.890		0.070	0.009 ± 0.006 (0.000–0.020)	–	–	–	0.451 ± 0.572 (0.140–1468)	–	–	–
Nitenpyram + *M. anisopliae*	1:1	24	y = 236.89x + 18.791		0.053	0.210 ± 0.168 (0.090–7820)	1.876	187.600	Synergistic	3.800 ± 0.859 (0.441–18.640)	1.707	170.730	Synergistic
		48	y = 254.43x + 41.593		0.001	0.031 ± 0.008 (0.002–0.050)	3.533	353.331	Synergistic	1.200 ± 0.802 (0.240–3136)	1.883	188.330	Synergistic
		72	y = 264.93x + 65.390		0.151	0.004 ± 0.003 (0.000–0.012)	2.250	225	Synergistic	0.121 ± 0.057 (0.071–1.730)	3.750	375	Synergistic
Dinotefuran		24	y = 100.02x + 8.720		0.040	0.806 ± 0.823 (0.260–7604)	–	–	–	6.59 ± 2.866 (0.850–4.422)	–	–	–
		48	y = 130.8x + 20.451		0.010	0.331 ± 0.216 (0.150–1995)	–	–	–	5.641 ± 0.548 (0.772–12.521)	–	–	–
		72	y = 138.82x + 39.700		0.190	0.076 ± 0.018 (0.020–0.171)	–	–	–	2.650 ± 0.153 (0.500–10.373)	–	–	–
Dinotefuran + *M. anisopliae*	1:1	24	y = 94.267x + 15.500		0.020	0.570 ± 0.547 (0.211–10.720)	1.414	141.4000	Synergistic	8.731 ± 19.301 (0.900–26.251	0.754	75.480	Antagonistic
		48	y = 125.71x + 36.561		0.080	0.100 ± 0.028 (0.05–2.95)	3.310	331	Synergistic	4.130 ± 0.788 (0.59–22.76)	1.365	136.5	Synergistic
		72	y = 94.267x + 15.500		0.001	0.020 ± 0.009 (0.004–0.040)	3.810	380	Synergistic	0.360 ± 0.176 (0.190–3.371)	7.361	736.1	Synergistic

*CTC* co-toxicity coefficient, *SF* synergy factor.

For dinotefuran, it was found that a combination with the EPF resulted in a synergistic interaction in all samples except for the LC_90_ at 24 h where antagonism was observed (SF = 0.754).

Pyriproxyfen showed synergistic interactions with *M. anisopliae* at all levels of data analysis (Table 4). The LC_50_ values of pyriproxyfen and *M. anisopliae* were 4.70, 1.04 and 0.18 ppm and LC_90_ values were 40.12, 9.13 and 7.83 ppm after 24, 48 and 72 h post exposure, respectively.

**Table 4:** Toxicity of insecticides (Pyriproxyfen and Spirotetramat) with and without *M. anisopliae* for *A. gossypii.*

Teatments	Ratio	Exposure period (hours)	Regression equation	Chi-square	LC_50_ ± SE (fiducial limits) (ppm)	SF	CTC	Type of action	LC_90_ ± SE (fiducial limits) (ppm)	SF	CTC	Type of action
Pyriproxyfen		24	y = 13.212x + 7.861	0.560	5.574 ± 0.618 (1.731–12.55)	–	–	–	40.350 ± 8.072 (5.030–204.1)	–	–	–
		48	y = 17.114x + 18.570	0.002	3.100 ± 0.300 (1.181–17.270)	–	–	–	54.332 ± 21.337 (5.390–91.060)	–	–	–
		72	y = 19.667x + 36.673	0.060	0.62 ± 0.178 (0.36–5.14)	–	–	–	23.940 ± 5.335 (3.541–27.920)	–	–	–
Pyriproxyfen + *M. anisopliae*	1:1	24	y = 13.102x + 10.923	0.010	4.701 ± 0.807 (1.580–2132)	1.185	118.500	Synergistic	40.122 ± 16.435 (5.070–85.02)	1.005	100.501	Synergistic
		48	y = 30.296x + 20.930	0.010	1.040 ± 0.305 (0.690–4.173)	2.980	298.070	Synergistic	9.530 ± 9.242 (2.951–1758.5)	5.700	570.090	Synergistic
		72	y = 22.695x + 49.060	0.030	0.184 ± 0.087 (0.000–0.321)	3.444	344.401	Synergistic	7.83 ± 9.709 (2.06–6581)	3.057	305.700	Synergistic
Spirotetramat		24	y = 40.771x + 6.280	0.060	1.791 ± 0.912 (0.522–13.02)	–	–	–	11.153 ± 2.596 (1.380–23.950)	–	–	–
		48	y = 59.693x + 16.271	0.001	1.000 ± 0.963 (0.363–11.070)	–	–	–	14.670 ± 3.280 (1.532–30.81)	–	–	–
		72	y = 70.884x + 32.142	0.040	0.260 ± 0.116 (0.141–5.280)	–	–	–	11.590 ± 2.129 (1.221–107.500)	–	–	–
Spirotetramat + *M. anisopliae*	1:1	24	y = 56.608x + 7.660	0.007	1.622 ± 0.870 (0.451–13.24)	1.104	110.400	Synergistic	13.14 ± 7.889 (1.48–25.20)	0.848	84.85	Antagonistic
		48	y = 94.698x + 20.743	0.171	0.360 ± 0.140 (0.220–3.551)	2.777	277.700	Synergistic	3.934 ± 4.729 (0.973–8866.600)	3.732	373.280	Synergistic
		72	y = 77.276x + 45.731	0.004	0.060 ± 0.027 (0.000–0.110)	4.333	433.300	Synergistic	3.790 ± 0.605 (0.721–22.010)	3.058	305.800	Synergistic

*CTC* co-toxicity coefficient, *SF* synergy factor.

Spirotetramat showed an antagonistic interaction with *M. anisopliae* for LC_90_ (CTC = 84.85, SF = 0.848) after 24 h, however, all other time points showed synergistic interactions (Table 4). For evaluation using the LC_50_, synergistic interactions were observed for all time points (SF > 1).

## Discussion

Insecticides have the potential to affect the various developmental stages of entomopathogenic fungi. The effect of an insecticide on conidial germination is the most important factor in determining fungus-insecticide compatibility^38,39^. We found that the insecticides tested did reduce vegetative growth and sporulation compared to the control but not always to the extent that would preclude compatibility of the insecticides tested, flonicamid, imidacloprid, nitenpyram, dinotefuran, pyriproxyfen, and spirotetramat exhibited good compatibility with *M. anisopliae*. Significantly reduced fungal colony diameter was observed for pymetrozine and matrine treatments. The insecticides caused different levels of inhibition of germination, vegetative growth, and sporulation of *M. anisopliae.* This is dependent on compounds present that block conidia metabolic functions as well as concentrations of the active compounds^40,41^. Oliveira^42^ reported that, molecules analogous to prosthetic groups diffuse to the cytoplasm where they bind to specific receivers affecting membrane permeability and enzymatic synthesis, consequently affecting metabolic processes. The same mechanism of inhibition is likely to be responsible for conidial germination and vegetative growth differences in *M. anisopliae*.

*M. anisopliae* have been employed effectively to control several insect pest species, including other aphid species such as *Lipaphis erysimi*^43^. Variation in interaction modalities (synergistic, antagonistic or neutral) of EPF with insecticides have been previously documented with species *B. bassiana* and *M. anisopliae*^44,45^. *A. gossypii* has developed high resistance to numerous common insecticides, such as neonicotinoids, carbamates, organophosphates, and pyrethroids^46–48^. Our study indicates that *M. anisopliae* has the potential to control *A. gossypii* within short period of time when combined with insecticides. The combined insecticide-*M. anisopliae* were consistently more toxic than individual treatments. Of the combinations tested, maximum mortality (91.68%) of *A. gossypii* was recorded with a mixture of flonicamid and *M. anisopliae* (2.4 × 10^6^ cfu/ml). Dayakar^49^ have previously found that the combination of insecticides with *M. anisopliae* can lead to a 1.19–1.42-fold increase in virulence over the sole treatment for Lepidoptera pests. The enhanced efficiency of combined application of fungal and chemical agents under laboratory conditions or field conditions has been reported in several studies^50,51^. Looking at the mustard aphid, *Lipaphis erysimi*, Purwar and Sachan^52^ also observed enhanced efficiency through an insecticide-EPF combination.

The present study utilized co-toxicity coefficients and synergy factors to calculate the efficacies of different insecticides + *M. anisopliae* formulations. The toxicity of insecticides, based on their LC_50_ and LC_90_ values increased when mixed with *M. anisopliae*. The mixture of insecticides and *M. anisopliae* as a 1:1 ratio demonstrates synergistic effects against *A. gossypii* (Tables 2, 3, 4) The antagonistic effect observed for imdiacloprid, dinotefuran, and spriotetramat at 24 h post exposure may be related to issues of compatibility, particularly suppression of EPF activity before the colony fully establishes, especially given that this antagonism is not observed at later time points. Ultimately, the combined treatments proved to be more effective than individual applications of all compounds tested (insecticides and *M. anisopliae*). The high values of co-toxicity coefficients, which were accompanied by insect mortalities > 90% for some treatments, illustrate the effectiveness of this dual-attack method of insect pest control. This finding is supported by previous studies, such as Quintela and McCoy^53,54^ which found that *B. bassiana* and *M. anisopliae* combined with sublethal doses of imidacloprid as a contact or oral treatment increased the mortality synergistically in the weevil, *Diaprepes abbreviatus*. Or the additive effect that has been observed with aphid species when *B. bassiana* is combined with a botanical pesticide, showing efficacy enhanced even in lower concentrations^55^.

From our findings we propose that dual modality approach is highly effective in achieving pest mortality. However, given the parity of compatibility of the insecticide with the EPF and its efficacy as a combined treatment, we identify that the insecticide’s direct effect on the EPF may be the primary criterion deciding success of a combination treatment.

## Conclusion

The combination of *M. anisopliae* with insecticides showed a synergistic effect and led to higher mortality of the cotton aphid, *A. gossypii*. If laboratory evidence for synergistic effects of *M. anisopliae* and insecticides against *A. gossypii* applies under greenhouse or field conditions, this control solution could mitigate potential issues related to environmental contamination, non-target impacts and pesticide resistance. However, further studies on the mechanism of toxicity of these combinations are needed.

## Data Availability

The data used and analyzed during this project are available from the corresponding author on reasonable request.

## Abbreviations

IPM: Integrated pest management
EPF: Entomopathogenic fungi
PDA: Potato dextrose agar
CFU: Colony forming unit
CTC: Co-toxicity coefficient
SF: Synergy factor
